# A flexible wearable sensor for knee flexion assessment during gait

**DOI:** 10.1016/j.gaitpost.2018.04.015

**Published:** 2018-05

**Authors:** Enrica Papi, Yen Nee Bo, Alison H. McGregor

**Affiliations:** a Department of Surgery and Cancer, Imperial College London, London, UK; b Department of Bioengineering, Imperial College London, London, UK

**Keywords:** Knee kinematics, Walking, Wearable technology, Gait analysis, Knee monitoring

## Abstract

**Background:**

Gait analysis plays an important role in the diagnosis and management of patients with movement disorders but it is usually performed within a laboratory. Recently interest has shifted towards the possibility of conducting gait assessments in everyday environments thus facilitating long-term monitoring. This is possible by using wearable technologies rather than laboratory based equipment.

**Research question:**

This study aims to validate a novel wearable sensor system’s ability to measure peak knee sagittal angles during gait.

**Methods:**

The proposed system comprises a flexible conductive polymer unit interfaced with a wireless acquisition node attached over the knee on a pair of leggings. Sixteen healthy volunteers participated to two gait assessments on separate occasions. Data was simultaneously collected from the novel sensor and a gold standard 10 camera motion capture system. The relationship between sensor signal and reference knee flexion angles was defined for each subject to allow the transformation of sensor voltage outputs to angular measures (degrees). The knee peak flexion angle from the sensor and reference system were compared by means of root mean square error (RMSE), absolute error, Bland-Altman plots and intra-class correlation coefficients (ICCs) to assess test-retest reliability.

**Results:**

Comparisons of knee peak flexion angles calculated from the sensor and gold standard yielded an absolute error of 0.35(±2.9°) and RMSE of 1.2(±0.4)°. Good agreement was found between the two systems with the majority of data lying within the limits of agreement. The sensor demonstrated high test-retest reliability (ICCs>0.8).

**Significance:**

These results show the ability of the sensor to monitor knee peak sagittal angles with small margins of error and in agreement with the gold standard system. The sensor has potential to be used in clinical settings as a discreet, unobtrusive wearable device allowing for long-term gait analysis.

## Highlights

• A wearable system to measure knee flexion is presented.
• A validation is conducted against a standard motion capture system.
• The novel system produced knee flexion angles in agreement with the gold standard.
• Knee flexion angle were estimated with small margins of errors.
• The novel system accuracy is comparable with systems described in the literature.

## 1 Introduction

The use of technology for gait analysis has led to improvements in gait assessment over standard observational analysis as it provides quantitative data on the gait cycle by objective measurement of body kinematics and kinetics [1]. With the introduction and recent development of wearable technologies, there is a growing interest in being able to transfer the analysis, usually performed in the laboratory, to real-life environments permitting long-term monitoring [2].

Knee angles are commonly reported as an outcome measures in the assessment of biomechanical function of our population both for clinical and research purposes. Patients affected by stroke, Parkinson’s disease, and osteoarthritis have abnormal knee flexion/extension patterns through the gait cycle [[3], [4], [5]]. The ability to monitor these kinematic changes can provide clinically important and relevant information to further our understanding of diseases progression as well as inform rehabilitation practice.

We previously developed a flexible conductive polymer sensor as knee sensing modality and used it in controlled knee movement condition to measure angles [6], to characterise exercises performance [7], classify activities of daily living and measure knee range of motion as surrogate of the sensor signal amplitude range in uncontrolled environments [8]. The aim of this study is to further validate the new sensor for measuring peak knee joint sagittal angles during gait.

## 2 Methods

### 2.1 Proposed knee sensor

**Fig. 1:**
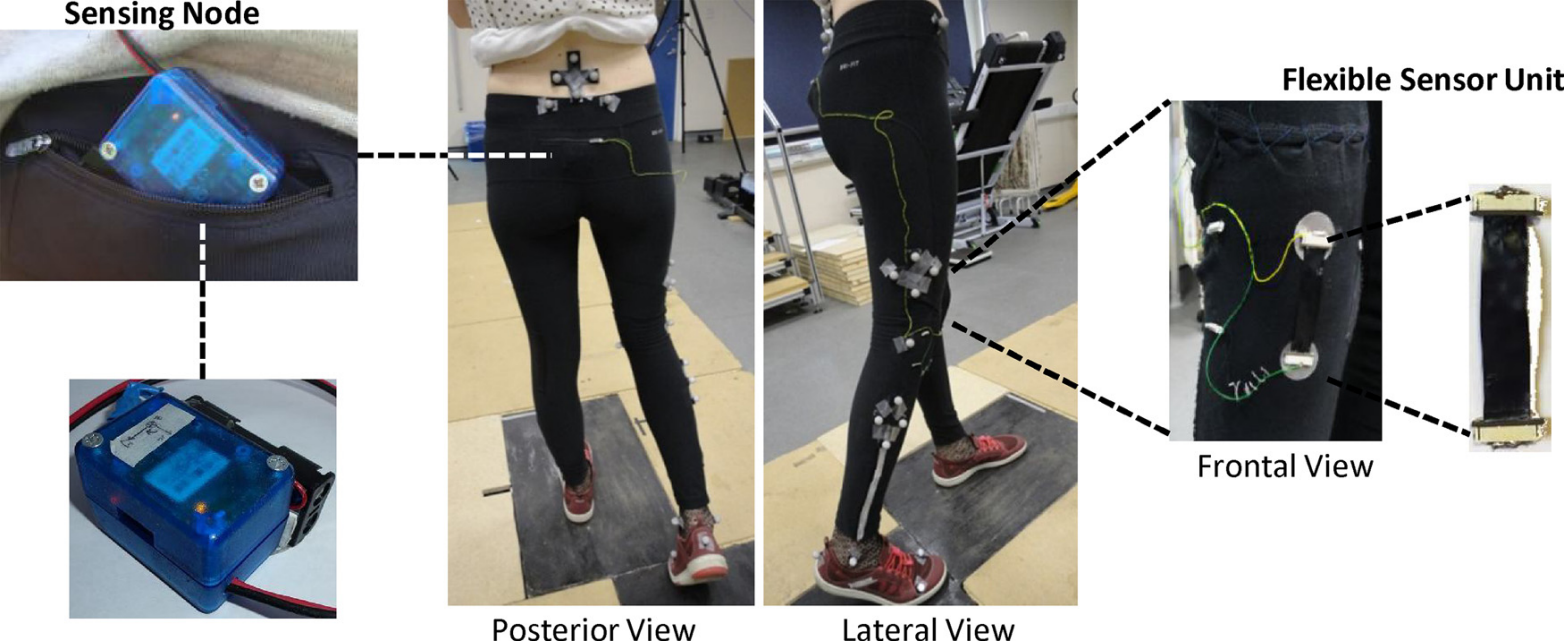
Markers and clusters positioning over the leggings, detail of the flexible sensor positioned anterior to the right knee and sensing node placed in the back pocket.

The sensor system consists of a flexible sensor unit and a sensing node for wireless data acquisition operated by 2 AA batteries [[6], [7], [8]]. The sensor unit (0.02 × 50 × 100 mm) is made by graphitized carbon black nanopowder (20%) embedded in a polyurethane substrate (80%). This material has a resistor like-function: as the sensor is stretched by knee movement, it changes resistance. The sensing node (40 × 50 × 35 mm) maps the resistance changes that occur by integrating the sensor signal to one arm of its Wheatstone bridge circuitry. It also contains a Bluetooth module (RN42, Microchip Technology Inc., Chandler, USA) that transmits data at 122 Hz to a laptop. A C++ interface allows real-time visualisation of the sensor signal. For this study, the sensor unit was positioned over the anterior aspect of the right knee on a pair of commercially available leggings (Fig. 1) by securing its two ends on them using super glue. The sensing node, wired to the unit, was positioned on the back pocket of the leggings.

### 2.2 Participants

Sixteen healthy participants (10 females, 6 males, age 23 ± 2.7 years, height 1.7 ± 0.8 m, Body Mass 64 ± 8.6 kg) were recruited in the study. Ethical approval was sought through the Imperial College Ethics Committee. All participants provided written informed consent prior to testing.

### 2.3 Experimental procedures

Participants attended two testing sessions with a week gap in between. In each session, participants were asked to perform one static trial and 10 walking trials at their preferred speed over a 6 m walkway. Data were acquired simultaneously from the sensor and a 10 camera motion capture system (MCS) operating at 100 Hz (Vicon, Oxford Metrics Ltd., UK). The marker set used comprised 8 individual markers attached on the pelvis and right lower limb anatomical landmarks (anterior and superior iliac spines, lateral and medial epicondyles, lateral and medial malleoli) and 3 four-marker clusters attached to the back and distally on the right thigh and shank segment. Markers and clusters were attached with double-sided tape over the leggings.

### 2.4 Knee angle calculation

Marker coordinates were filtered using Woltring’s general cross-validatory quintic smoothing spline with a predicted mean square error of 15 mm [9]. Anatomical frames of reference from the markers 3D coordinates were defined in accordance with previous recommendations for the right hip, knee and ankle [10]. 3-D knee angles were calculated based on the joint coordinate system convention [11]. Only sagittal knee angles were considered for further analysis.

Sensor data were filtered using a 4th order Butterworth filter with 10 Hz cut-off frequency.

MCS and sensor data were time normalised to 100% of the gait cycle. To estimate knee sagittal angles directly from the proposed sensor the relationship between sensor signal (mV) and MCS knee angles (°) was sought to obtain the transformation function from voltage to degrees. A function for each participant was defined during the first test session. Data from the 5th trial were used for defining the transformation function to account for any sensor adjustments that may have occurred following the first trials. The function was obtained through a linear fit of sensor and MCS data from one walking trials and applied to sensor outputs of the remaining 9 trials and data of the second test. The peak knee flexion angles calculated from the sensor and from MCS data for 9 trials of each test session were averaged and compared. Data analysis was performed using Matlab (MathWorks Inc., Natick, USA).

### 2.5 Statistics

Descriptive statistics (mean and standard deviation) were used to summarise the results. Correlation (r^2^) and the level of agreement (Bland-Altman method) between the two approaches were evaluated. The absolute difference and root mean square errors (RMSEs) were computed to determine the robustness and accuracy of the sensor angles. Test-retest reliability was assessed using intra-class correlation coefficients. Statistical analyses were performed using Matlab and SPSS (SPSS Inc., Chicago, USA).

## 3 Results

**Fig. 2:**
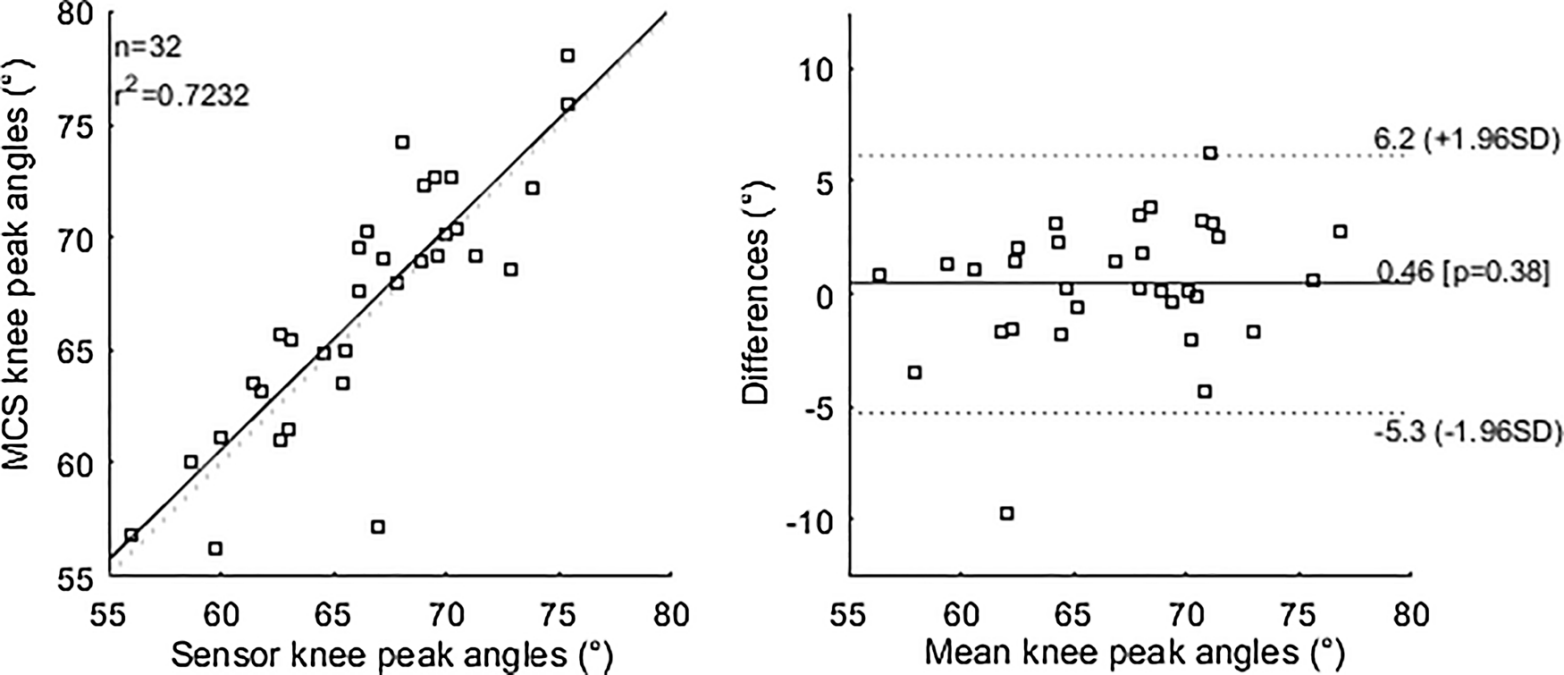
Correlation and Bland Altman plot of agreement between knee peak angles measured by the sensor and MCS. Horizontal lines represent the mean difference and limits of agreement (dotted lines).

The mean sensor and MCS knee peak flexion angles were 66.3(±5.8)° and 66.9(±5.1)° for test 1 and 66.9(±4.8)° and 66.2(±4.6)° for test 2. Values for all participants during both testing sessions are shown in Table 1. A correlation coefficient of 0.7 was found when combining the mean from all subjects and both tests (Fig. 2a). Fig. 2b is the Bland-Altman plot showing a high level of agreement between the two approaches with the majority of the data point within the locus of agreement. 95% limits of agreement and the mean difference are shown in the plot. A mean absolute error of 0.07(±3.3)° and −0.8(±3.3)° were obtained for test 1 and 2 respectively. A mean RMSE of 1.2(±0.4)° was found for both tests. Values for each participant are reported in Table 1. High test-retest reliability was observed with ICC values above 0.8 for the knee sensor and 0.9 for MCS for all participants.

**Table 1:** Results for all participants during two test sessions. Standard deviation is shown in brackets.

	Test 1				Test 2			
Participants	Mean Sensor Peak Angle (°)	Reference MCS Peak Angle (°)	Average Difference (°)	RMSE (°)	Mean Sensor Peak Angle (°)	Reference MCS Peak Angle (°)	Average Difference (°)	RMSE (°)
1	70.2 (4.4)	70 (1.6)	−0.16 (4.4)	1.75	65.7 (1.8)	62.7 (1.1)	−3.07 (1.61)	1.07
2	70.4 (3.3)	70.5 (1.1)	0.12 (2.9)	1.05	69.0 (4.0)	68.8 (1.3)	−0.16 (3.73)	1.28
3	56.8 (2.2)	55.9 (1.2)	−0.84 (2.0)	1.18	63.1 (2.0)	61.75 (1.1)	−1.39 (1.54)	0.88
4	78. 1 (3.6)	75.4 (2.1)	−2.73 (2.9)	1.76	72.3 (8.2)	68.9 (1.7)	−3.27 (7.8)	1.55
5	63.5 (6.2)	65.4 (1.1)	1.85 (7.1)	0.66	75.97 (4.6)	75.3 (0.9)	−0.64 (4.23)	0.85
6	63.5 (3.1)	61.4 (1.6)	−2.07 (2.2)	1.13	60.9 (7.6)	62.6 (1.4)	1.63 (7.7)	1.02
7	72.2 (1.3)	73.8 (1.0)	1.63 (1.7)	1.11	68.6 (2.8)	72.9 (0.7)	4.3 (3.14)	0.85
8	56.2 (4.7)	59.7 (1.3)	3.5 (4.1)	0.21	60.1 (2.9)	58.7 (1.1)	−1.37 (2.17)	0.62
9	72.7 (3.0)	70.2 (2.1)	−2.56 (2.2)	1.64	68.0 (1.9)	67.8 (1.2)	−0.24 (2.55)	1.25
10	57.2 (4.8)	66.9 (1.0)	9.7 (4.6)	0.97	69.2 (7.1)	71.3 (2.2)	2.03 (5.84)	1.71
11	72.7 (3.2)	69.5 (1.4)	−3.17 (2.6)	1.17	67.6 (3.5)	66.1 (1.7)	−1.49 (3.68)	1.81
12	65.5 (3.0)	63.2 (1.7)	−2.33 (2.0)	1.7	61.1 (2.7)	60.0 (1.2)	−1.09 (2.59)	1.24
13	70.3 (5.2)	66.51 (5.1)	−3.80 (9.6)	1.51	61.49 (2.6)	63.0 (1.2)	1.53 (1.91)	0.85
14	64.8 (3.3)	64.6 (0.9)	−0.23 (2.7)	0.93	74.2 (4.5)	68.0 (2.7)	−6.21 (2.5)	1.71
15	69.0 (3.3)	67.2 (1.0)	1.85 (3.4)	1.08	64.9 (2.8)	65.5 (1.9)	0.59 (1.5)	1.03
16	69.2 (2.2)	69.6 (0.7)	0.43 (2.2)	0.82	69.6 (3.7)	66.1 (2.1)	−3.4 (3.1)	2.07
Mean	66.3	66.9	0.07	1.2	66.9	66.2	−0.8	1.2
SD	5.8	5.1	3.3	0.4	4.9	4.7	3.3	0.4

MCS:Motion Capture System; RMSE:root mean square error.

## 4 Discussion

This study aimed to validate the proposed knee system in measuring knee sagittal angles. Knee peak flexion angles could be calculated from the sensor signal output by applying equations derived from linear fittings of MCS angles and sensor outputs for each participant. A small bias (0.46°) and good agreement were found between the sensor and MCS as from Bland-Altman analysis. Moreover, a substantial correlation was observed and the sensor demonstrated also excellent repeatability based on coefficients interpretation according to Landis & Koch [12]. The RMSE values obtained are comparable to those reported in the literature (range between 1.3° and 6.8°) when using inertia measurement units or accelerometers combined with magnetometers or string sensors [[13], [14], [15], [16]]. Furthermore, in comparison to the other systems, the proposed one relies only on one small sensor unit against the need for multiple sensors. This makes our system advantageous to use for unobtrusive and discreet measurements in everyday life settings. Moreover, in addition to a similar sensor [16], our system has been previously validated to identify activities of daily living and exercise performance [[7], [8]]; this combined with its ability to measure peak flexion angles, makes our sensor capable of providing a more comprehensive description of subjects’ movement capabilities for clinical use. One limitation of the sensor is that requires a subject-specific calibration requiring a motion capture system. Moreover, we found that the sensor gave inaccurate outputs at low range of the knee angle and thus we concentrated this preliminary analysis to peak knee flexion. Future work should be directed to investigate a generalise method to determine knee angles from the sensor signal, calculate knee angles over time and finalise sensor design based on patients’ preferences [17].

## Conflict of interest statement

There were no conflicts of interest in this study.
